# Descriptive study of COVID-19 outbreak among passengers and crew on Diamond Princess cruise ship, Yokohama Port, Japan, 20 January to 9 February 2020

**DOI:** 10.2807/1560-7917.ES.2020.25.23.2000272

**Published:** 2020-06-11

**Authors:** Takuya Yamagishi, Hajime Kamiya, Kensaku Kakimoto, Motoi Suzuki, Takaji Wakita

**Affiliations:** 1 Infectious Disease Surveillance Center, National Institute of Infectious Diseases, Tokyo, Japan; 2 Field Epidemiology Training Program, National Institute of Infectious Diseases, Tokyo, Japan; 3 National Institute of Infectious Diseases, Tokyo, Japan

**Keywords:** COVID-19, novel coronavirus, cruise ship, Diamond Princess, quarantine

## Abstract

An outbreak of coronavirus disease (COVID-19) occurred on the Diamond Princess cruise ship making an international journey, which led to quarantine of the ship at Yokohama Port, Japan. A suspected COVID-19 case was defined as a passenger or crew member who developed a fever or respiratory symptoms, and a confirmed COVID-19 case had laboratory-confirmation of severe acute respiratory syndrome coronavirus 2 (SARS-CoV-2) infection. Between 3 and 9 February 2020, 490 individuals were tested for SARS-CoV-2 and 172 were positive (152 passengers (median age: 70 years; interquartile range (IQR): 64–75; males: 45%) and 20 crew (median age: 40 years; IQR: 35–48.5; males: 80%). Other than the Hong Kong-related index case, symptom onset for the earliest confirmed case was 22 January, 2 days after the cruise ship left port. Attack rates among passengers were similar across the decks, while beverage (3.3%, 2/61) and food service staff (5.7%, 14/245) were most affected. Attack rates tended to increase with age. A comprehensive outbreak response was implemented, including surveillance, provision of essential medical care, food and medicine delivery, isolation, infection prevention and control, sampling and disembarkation.

## Background

On 7 January 2020, Chinese health officials announced detection of a case with novel coronavirus disease (COVID-19) in Wuhan, China [1,2]. As the outbreak expanded in Wuhan, there was spillover of COVID-19 cases outside the country. The first COVID-19 cases outside of China were reported in Thailand on 13 January [3]. Now, all World Health Organization (WHO) Regions have domestic cases and the number of cases has been increasing in many countries in Europe and the Americas [4]. Japan detected its first COVID-19 case on 14 January 2020, a Japanese resident who travelled to Wuhan for winter vacation and returned to Japan for work [5]. There were subsequent reports of new cases in Japan both with and without travel history to Wuhan [6].

The reason for the high speed of COVID-19 expansion is still under debate, but transmission from asymptomatic cases or pre-symptomatic is suggested [7]. For other betacoronaviruses, such as severe acute respiratory syndrome coronavirus (SARS-CoV) and Middle East Respiratory Syndrome coronavirus (MERS-CoV), the primary mode of transmission is considered to be through droplets or direct contact of the mucous membranes with infectious respiratory droplets or fomites [8,9]. The primary mode of transmission for SARS-CoV-2 is also considered to be via droplets, along with contribution from contact [10]. However, SARS-CoV has exhibited efficient transmission, leading to a higher-than expected number of cases (super-spreading event) [8], with possible airborne transmission being observed under particular conditions [11].

Here, we report our preliminary findings from the investigation of a COVID-19 outbreak on an international cruise ship, the Diamond Princess, at Yokohama Port, Japan up to 9 February 2020. The findings from this descriptive study may facilitate increased understanding of COVID-19 and could contribute to public health responses on how to address cruise-related COVID-19 outbreak challenges.

## Outbreak detection

A case of COVID-19 related to the internationally-operated Diamond Princess cruise ship was reported to the Ministry of Health, Labour and Welfare (MHLW), Japan on 1 February by health officials in Hong Kong [12]. Japan was notified by Hong Kong authorities on 2 February that the Hong Kong-related COVID-19 case (index case) had arrived in Japan on 17 January and boarded the cruise ship on 20 January at Yokohama, Japan. The case participated in a tour of Kagoshima, Japan on 22 January during a shore-based stop and disembarked the ship in Hong Kong on 25 January. The case had a cough starting on 19 January, developed a fever on 30 January and was diagnosed with COVID-19 at a hospital in Hong Kong. The cruise-ship implemented a quarantine on 3 February, after which 10 laboratory-confirmed cases of COVID-19 were identified on board on 4 February.

## Methods

### Setting

The cruise ship had 2,645 passengers and 1,068 crew members onboard from more than 50 countries. There were eight decks for passenger cabins and eight decks for crew cabins. The ship contained a seven-bed medical clinic staffed by doctors and nurses. After the cruise ship left Yokohama Port, Japan on 20* January, it visited Hong Kong on 25 January, Vietnam on 27 January, Taiwan on 31 January and Okinawa, Japan on 1 February, returning to Yokohama Port on 3 February.

### Definition of cases and close contacts

We defined a confirmed case as a passenger or crew member with positive RT-PCR test result for SARS-CoV-2 (Box). We defined a suspected case as a passenger or a crew member who had a fever ≥ 37.5 °C or respiratory symptoms during the investigation period or identified during the review of the ship’s medical clinic records. An asymptomatic case was defined as a person without symptoms at the time of investigation. We defined a close contact as someone who joined the Kagoshima tour with the index case, who shared a cabin with a confirmed case or who shared the same cabin with a suspected case.

Box **Definitions of confirmed and suspected cases, and close contacts in Diamond Princess cruise ship COVID-19 outbreak, Yokohama Port, Japan, 20 January–9 February 2020**

**Confirmed case:** Passengers or crew who tested positive for severe acute respiratory syndrome coronavirus 2 (SARS-CoV-2) by RT-PCR

**Suspected case:** Passengers or crew who had a fever  ≥ 37.5 °C or respiratory symptoms

**Close contact:**

- Passengers or crew who joined a tour of Kagoshima, Japan

- Passengers or crew who shared the same cabin with a confirmed case

- Passengers or crew who shared the same cabin with a suspected case^a^

^a^ The third definition was only used until 6 February 2020, when the operation was strained by increasing number of suspected and confirmed cases.

### Testing strategy

Starting on 3 February, quarantine officers took oropharyngeal samples from suspected cases and close contacts. Samples were tested for SARS-CoV-2 using a RT-PCR test according to the national standard [13]. Testing was conducted by local public health laboratories in Yokohama and neighbouring areas, and quarantine laboratories at Yokohama, Narita, and Tokyo. As at 7 February, we changed the testing strategy and only tested suspected cases, which had been increasing in number. Along with the change in testing strategy, starting from 7 February, a hotline for self-identified febrile passengers and crew was set up and a fever clinic was launched alongside the ship’s medical centre. Following a call to the hotline, doctors from Japan’s disaster medical assistance team (DMAT) and Ministry of Defence went to the cabin of the individual to assess their condition. After evaluation, an oropharyngeal swab was taken from suspected cases for RT-PCR testing.

### Data collection

We collected all suspected and confirmed cases’ general information, clinical symptoms, RT-PCR test results and contact information from the multiple sources described in Table 1. We merged the data to create a line list for the period of 3 to 9 February.

**Table 1:** Data sources during investigation of Diamond Princess cruise ship COVID-19 outbreak, Yokohama Port, Japan, 20 January–9 February 2020

Source	Time period reviewed	Notes
Medical consultation records at the ship’s medical centre	20 January–6 February	Data from the medical consultation records between 20 January, when the ship started the cruise, and 6 February when the temporary fever clinic was set up on the ship. Reviewed for assessing suspected cases.
RT-PCR test results	3­–9 February	All RT-PCR test results were collected from quarantine laboratories.
Consent form for laboratory testing	3–9 February	Used to collect demographic information, as well as symptoms and symptom onset date via paper-based questionnaire with consent form.
Fever hotline and medical records of on-board fever clinic	8–9 February	The log of fever hotline and medical charts from the fever clinic were reviewed for assessing suspected cases.

### Data analysis

We described the epidemiology of the suspected cases and confirmed cases. We calculated the test positivity rate among suspected cases according to the date of the sample. The attack rate was calculated based on the passenger and crew data provided by the cruise ship. We used Excel to conduct all the analysis.

### Ethical statement

This report was exempt from the requirement for institutional ethics review since it was a public health investigation approved by the Japanese Infectious Disease Law and Quarantine Law.

## Results

A total of 490 samples, representing 490 individuals, were tested for SARS-COV-2 between 3 and 9 February. Of these, 358 (73%) were suspected cases, 86 (18%) were close contacts, two were individuals tested before emergency disembarkation for medical reasons, three were family members of these individuals and the remainder (n = 41, 8%) were individuals without information about symptoms and contacts. Of the total 490 samples tested, 172 (35%) were positive. Among the 172 confirmed cases, 144 (84%) met the suspected case definition before testing while 19 (11%) shared a cabin with a confirmed case (Table 2). Among the 172 confirmed cases, 87 (51%) cases were female and 85 (49%) were male. Passenger cases had a higher median age (70 years; interquartile range (IQR): 64–75) compared with crew cases (40 years; IQR: 35–48.5). Among the 168 confirmed cases with information about symptoms at the time of sampling available, 24 were asymptomatic (14%); including the two individuals who disembarked because of emergency medical indication. Among symptomatic cases (n = 144), the most common symptom was fever ≥ 37.5 °C (n = 121, 84%), followed by cough (n = 69, 48%). Three cases mentioned diarrhoea as a symptom, which was less common than sore throat.

**Table 2:** Characteristics of confirmed COVID-19 cases in Diamond Princess cruise ship outbreak, Yokohama Port, Japan, 20 January–9 February 2020 (n = 172)

Characteristic	Number (n)	Proportion (%)
Female	87	51
Male	85	49
Median age (years) (IQR)	69 (60–74)	
**Nationality**		
Japanese	80	47
Non-Japanese	92	53
**Screened for novel coronavirus**		
Suspected cases	144	84
Close contacts	19	11
Before disembarking for medical care^a^	2	1
Other^b^	7	4
**Presence on ship**		
Passengers	152	88
- Median age (years) (IQR)	70 (64–75)	
- Female	83	55
- Male	69	45
Crew	20	12
- Median age (years) (IQR)	40 (35–48.5)	
- Female	4	20
- Male	16	80
**Symptoms at time of sampling**		
Symptomatic	144	84
- Fever	121	84^c^
- Cough	69	48^c^
- Sore throat	20	14^c^
- Diarrhoea	3	2^c^
Asymptomatic	24	14
Not available	4	2

IQR: interquartile range.

^a^ Two were passengers who were tested before disembarking for emergency medical care.

^b^ Includes three family members accompanying the two passengers who disembarked and four passengers without information about symptoms and contacts.

^c^ Proportion among 144 symptomatic cases.

**Figure 1:**
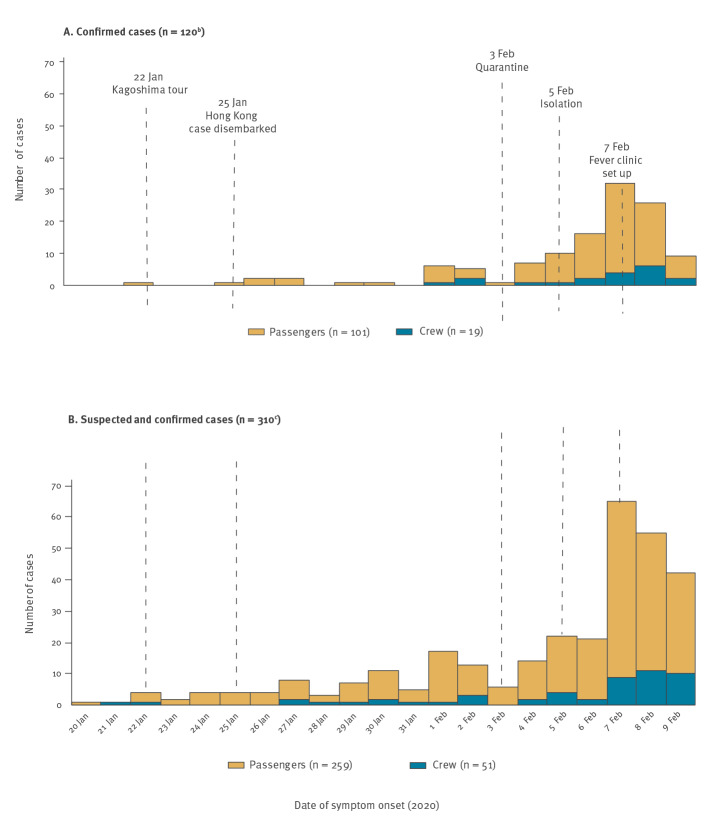
Symptom onset date^a^ for confirmed (A) and suspected (B) COVID-19 cases in Diamond Princess cruise ship outbreak, Yokohama Port, Japan, 20 January–9 February 2020

^a^ Symptom onset date was defined as the onset of a fever  ≥ 37.5 °C or respiratory symptoms.

^b^ Of 172 confirmed cases, 144 were known to have symptoms but date of onset was not available for 24 cases, including 1 crew member and 23 passengers.

^c^ Of 358 suspected cases, date of onset was not available for 48 cases.

The earliest date of symptom onset among the confirmed cases onboard was 22 January (Figure 1A). The number of confirmed cases remained low for approximately 2 weeks after, and then showed a notable increase. Symptom onset date of the first crew member case was 1 February, 10 days after that of the first passenger case. The epidemic curve of suspected cases indicated that passengers and crew members were exhibiting symptoms from the beginning of the cruise and the number of suspected cases gradually increased daily (Figure 1B). The cumulative incidence of suspected cases between 20 January and 2 February, i.e. prior to quarantine on 3 February, was 1.92 per 1,000 passengers and 0.87 per 1,000 crew members between 20 January and 2 February.

The index case developed a cough on 19 January, joined a tour of Kagoshima, Japan on 22 January, disembarked in Hong Kong on 25 January and developed a fever on 30 January in Hong Kong.

**Figure 2:**
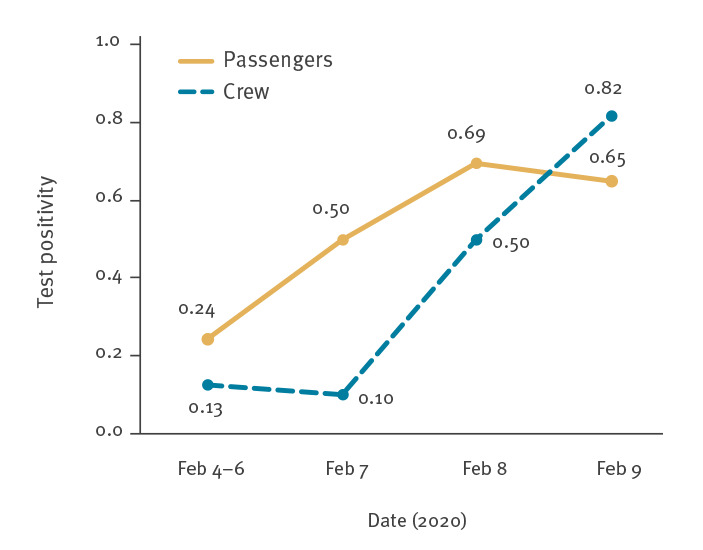
SARS-COV-2 test positivity rates for suspected COVID-19 cases^a^ in Diamond Princess cruise ship outbreak, Japan, 4–9 February 2020 (n = 358)

^a^ Passengers or crew who had a fever  ≥ 37.5 °C or respiratory symptoms.

Test positivity by testing date among suspected cases was 24% (36/148) from 4 to 6 February and increased to 69% (52/75) on 8 February for passengers whereas it was one of eight on 4 to 6 February and nine of 11 on 9 February for crew members (Table 3, Figure 2).

**Table 3:** SARS-COV-2 test positivity rates for suspected COVID-19 cases^a^ in Diamond Princess cruise ship outbreak, Japan, 4–9 February 2020 (n = 358)

Sampling date	Feb 4-6		Feb 7		Feb 8		Feb 9		Total	
	Positive/tested	%	Positive/tested	%	Positive/tested	%	Positive/tested	%	Positive/tested	%
Passengers	36/148	24	1/2	50	52/75	69	35/54	65	124/279	44
Crew	1/8	13	5/50	10	5/10	50	9/11	82	20/79	25
Total	37/156	24	6/52	12	57/85	67	44/65	68	144/358	40

^a^ Passengers or crew who had a fever  ≥ 37.5 °C or respiratory symptoms.

The attack rate was highest among passengers 20 to 29 years of age, but this is based on small numbers. The attack rates by age group among passengers were lowest in the 40 to 49 years group and increased with age thereafter (Table 4). Attack rates among crew also tended to increase with age. The attack rate of passengers by deck was relatively similar for each deck, except deck F where no cases were reported during our investigation period. On the other hand, most of the crew member cases were on deck 3, where the restaurant staff mainly had cabins. Accordingly, the attack rate of restaurant staff was the highest among the crew cases (Table 4).

**Table 4:** COVID-19 attack rates of passengers and crew members by deck, workplace and age in Diamond Princess cruise ship outbreak, Yokohama Port, Japan, 20 January–9 February 2020 (n = 2,645 passengers, n = 1,068 crew)

Age, deck, workplace	Passengers			Crew members		
	Confirmed (n)	Total (n)	Percentage (%)	Confirmed (n)	Total (n)	Percentage (%)
**Age group (years)**						
≤ 19	0	38	0.0	0	1	0.0
20–29	5	45	11.1	4	302	1.3
30–39	2	49	4.1	5	381	1.3
40–49	1	69	1.4	6	265	2.3
50–59	14	300	4.7	5	98	5.1
60–69	48	903	5.3	0	21	0.0
70–79	63	1,013	6.2	0	0	NA
≥ 80	19	228	8.3	0	0	NA
Total	152	2,645	5.7	20	1,068	1.9
**Deck**						
L	8	132	6.1	NA	NA	NA
A	35	463	7.6	NA	NA	NA
B	19	536	3.5	NA	NA	NA
C	29	521	5.6	NA	NA	NA
D	24	435	5.5	NA	NA	NA
E	26	373	7.0	NA	NA	NA
F	0	27	0.0	NA	NA	NA
P	11	158	7.0	NA	NA	NA
2	NA	NA	NA	2	171	1.2
3	NA	NA	NA	16	582	2.7
4	NA	NA	NA	1	148	0.7
5	NA	NA	NA	1	84	1.2
6	NA	NA	NA	0	33	0.0
7	NA	NA	NA	0	30	0.0
12	NA	NA	NA	0	6	0.0
14	NA	NA	NA	0	12	0.0
Total	152	2,645	5.7	20	1,066^a^	1.9
**Crew workplace**						
Beverage service	NA	NA	NA	2	61	3.3
Housekeeping	NA	NA	NA	1	176	0.6
Food service (restaurant)	NA	NA	NA	14	245	5.7
Steward	NA	NA	NA	1	53	1.9
Other	NA	NA	NA	2	533	0.4
Total	NA	NA	NA	20	1,068	1.9

NA: not applicable.

^a^ Deck information for two crew members was not available.

## Outbreak control measures

After notification of the case from Hong Kong, the MHLW began a quarantine on the cruise ship at Yokohama Port on 3 February. The Emergency Operating Centre (EOC) was set up on 5 February, and on this date, all passengers were also isolated in their cabins and internal air re-circulation was stopped to reduce the possible risk of airborne transmission. Crew members continued to work, following guidance from the ship’s standard health precautions, and were isolated when individual crew members were considered to be infectious after assessment by the cruise ship’s medical staff.

On 7 February, a temporary on-site fever clinic was launched to address the increasing number of febrile passengers and crew members by Japanese medical staff, while the ship’s medical clinic focused on other patients, those with non-COVID-19-related symptoms, on the cruise ship. Respiratory samples were taken daily from suspected cases and were shipped to the quarantine laboratories for testing once or twice a day. The laboratory results came the following day and all confirmed cases were disembarked and transferred to designated hospitals within a day.

MHLW provided medicine, surgical/N95 masks, gloves, alcohol-based hand rub, and basic knowledge of coronavirus infection and infection prevention to passengers (e.g. hand hygiene and cough etiquette). It also provided training on infection prevention and control to the crew. Quarantine personnel wore surgical masks, except during meals at the EOC on the cruise ship, with added eye protection, long-sleeve gowns and gloves when taking samples. MHLW decided to disembark all the asymptomatic passengers and crew members, and those with symptoms who tested negative after 14 days of quarantine. Because of the mass testing workload, MHLW prioritised testing populations, with individuals over 80 years of age prioritised for sampling before disembarkation.

The outbreak response operation was complex and we worked closely with the captain, managers and crew of the cruise ship to make decisions for the next steps in response efforts. The primary challenge identified was food preparation and delivery to quarantined passengers. Because passengers needed to be isolated, we requested the crew to continue to work, so meals were prepared and delivered by crew members under the guidance of infection prevention and control staff. Another challenge was the language barrier which made it difficult to conduct effective isolation measures because of the delay in providing instruction; however, collaboration with crew members worked efficiently.

## Discussion

This report details the early phase of the outbreak investigation on a cruise ship quarantined in Yokohama Port that followed the confirmation of a disembarked passenger having COVID-19. This event required a large-scale quarantine that we had not experienced before, with a large number of international passengers and crew members further adding to the public health challenge. We believe that the findings from our experience are useful to respond to a similar COVID-19 event in an international cruise ship such as that quarantined at California in March 2020 [14] or Nagasaki in April 2020 [15].

The earliest date of symptom onset for a confirmed case was 22 January, which was only 2 days after the start of the voyage and which suggests that the case could have already been infected before the voyage. The outbreak of COVID-19 had already been reported in Wuhan by mid-January, and so it was also possible that more than one COVID-19 case might have been on board the cruise ship. It is also reasonable to infer that the index case became infectious while onboard the cruise ship. The epidemic curve of symptomatic cases showed that passengers and crew members with fever and/or respiratory illness increased immediately after the start of the cruise. There were several well-attended cocktail and wine parties during the cruise where large numbers of passengers gathered at one place for several hours. Even though passengers had no symptoms when they attended the party, possible transmission of SARS-CoV-2 from asymptomatic or pre-symptomatic cases has been reported [16,17]. Attendance at these parties could explain why passenger cases were scattered across all the decks with no discernible pattern. Also, further, continued exposure to the virus can be assumed to have ended when passengers were isolated in their cabins, unless a cabin mate was already infected. Some confirmed cases in cabin mates were identified soon after the start of isolation on 5 February, suggesting that they were exposed to the virus before the isolation began. The effectiveness of isolation measures should be evaluated at least 2 weeks after the initiation of isolation, since cases identified later need to be counted.

The results suggest that the transmission dynamics among crew was different from those among passengers. Among passengers, the highest attack rate was observed in the 20 to 29 years of age group, and this might be because of frequent contacts among young people, which could be a driving force of the disease’s spread. The only interaction between crew and passengers was during crew work shifts, as crew members spent most of their time in a relatively fixed place in their crew quarters. The attack rate among food service workers was higher than among other occupations among the crew (Table 2), which supports the hypothesis that the disease spread through cocktail and wine parties. The possibility of airborne transmission of SARS-CoV-2 could not be ruled out at that time [18] and internal air re-circulation was stopped on the cruise ship. Our findings suggest that SARS-CoV-2 transmission among close contacts (droplet and direct contact) as opposed to airborne transmission, was primarily responsible for COVID-19’s spread on the ship.

Passengers and crew aged over 50 years were more likely to develop symptoms, especially individuals over 80 years of age. Because older people were at higher risk of developing severe infections [19], they were the population to be prioritised for testing and disembarkation. Another high-risk population for poor health outcomes were those with a comorbidity; however, it took additional time to check for comorbidities among passengers on the cruise ship.

We detected 24 asymptomatic cases (14%) among confirmed cases, mainly among passengers who joined a bus tour in Kagoshima. It is important to note that being asymptomatic at one point of time does not exclude the possibility of developing symptoms later on [16,20]. The two cases who disembarked the ship because of another medical indication were positive for COVID-19 but asymptomatic at the time of disembarkation. This finding led us to recommend that all passengers be tested regardless of their symptoms. The recent finding that the viral load of SARS-CoV-2 in asymptomatic cases was reported to be similar to that in symptomatic cases supports our judgment [21]. This poses the challenge for contact tracing and isolation measures that are usually considered after symptom onset.

The increasing test positivity rate among the crew was a worrying sign even though most were based on small numbers. Test positivity is used as a surrogate of incidence [22], and the incidence among the crew was considered to be high. Because the crew were vital to continue ship’s operations and the provision of food to more than 3,000 individuals onboard the ship, stopping their contact with others would have limited vital services to passengers and cruise ship operations. The decision to request that the crew continue ship’s operations was a highly political one.

The strength of this study includes the availability of a classic cohort and information from all passengers and crew onboard the ship. Two main limitations of this report are sampling and reporting biases. Under the emergency response for a novel infectious disease, it was difficult to test the entire target population because of limited testing capacity. As a result, we changed our strategy to test cabin mates of positive cases only if they met the criteria of suspected cases. Also, crew had to continue their daily routine along with tasks related to the quarantine, and fever may not have been reported comprehensively. Finally, we tried to identify the incubation period by evaluating cabin mates of the confirmed cases. However, it was impossible to distinguish the source of the infection as being the cabin mate or an exposure before the isolation.

## Conclusion

We summarised the epidemiology of an early phase of a COVID-19 outbreak on a cruise ship. The outbreak started shortly after the start of the journey of the cruise ship and among passengers, it seemed to be controlled by the isolation in their cabin. The decision to continue to have crew working was a political one aiming to ensure the provision of ongoing passenger services and ship operations during quarantine at a time when there was a concurrent need to limit further transmission on board the ship. A quarantine operation on a large cruise ship requires a comprehensive outbreak response including a high-level of collaboration with crew members.

## *Authors’ correction

A mistake in the date the cruise ship left Yokohama Port, Japan was corrected on 12 June 2020, upon request of the authors.

## References

[1] Huang C Wang Y Li X Ren L Zhao J Hu Y Clinical features of patients infected with 2019 novel coronavirus in Wuhan, China. Lancet. 2020;395(10223):497-506. 10.1016/S0140-6736(20)30183-5 31986264 PMC7159299

[2] Zhu N Zhang D Wang W Li X Yang B Song J A novel coronavirus from patients with pneumonia in China, 2019. N Engl J Med. 2020;382(8):727-33. 10.1056/NEJMoa2001017 31978945 PMC7092803

[3] Pongpirul WA Pongpirul K Ratnarathon AC Prasithsirikul W Journey of a Thai taxi driver and novel coronavirus. N Engl J Med. 2020;382(11):1067-8. 10.1056/NEJMc2001621 32050060 PMC7121480

[4] World Health Organization (WHO). Situation report – 141 Coronavirus disease 2019 (COVID-19) 9 June 2020. Geneva: WHO. [Accessed: 10 Jun 2020]. Available from: https://www.who.int/emergencies/diseases/novel-coronavirus-2019/situation-reports/

[5] Ministry of Health, Labour and Welfare. [The first case of novel coronavirus infection in Japan. Tokyo: Ministry of Health, Labour and Welfare]. [Accessed: 8 Mar 2020]. Japanese. Available from: https://www.mhlw.go.jp/stf/newpage_08906.html

[6] National Institute of Infectious Diseases (NIID). [Descriptive Epidemiology of 12 Confirmed 2019-Novel Coronavirus (2019-nCoV) Cases in Japan (as of 3 February 2020)]. Tokyo: NIID; 2020. Japanese. Available from: https://www.niid.go.jp/niid/en/2019-ncov-e/2488-idsc/iasr-news/9400-481pe01.html

[7] Furukawa NW Brooks JT Sobel J Evidence Supporting Transmission of Severe Acute Respiratory Syndrome Coronavirus 2 While Presymptomatic or Asymptomatic. Emerg Infect Dis. 2020;26(7). 10.3201/eid2607.201595 32364890 PMC7323549

[8] Peiris JSM Yuen KY Osterhaus AD Stöhr K The severe acute respiratory syndrome. N Engl J Med. 2003;349(25):2431-41. 10.1056/NEJMra032498 14681510

[9] Arabi YM Balkhy HH Hayden FG Bouchama A Luke T Baillie JK Middle East Respiratory Syndrome. N Engl J Med. 2017;376(6):584-94. 10.1056/NEJMsr1408795 28177862 PMC5362064

[10] van Doremalen N Bushmaker T Morris DH Holbrook MG Gamble A Williamson BN Aerosol and surface stability of SARS-CoV-2 as compared with SARS-CoV-1. N Engl J Med. 2020;382(16):1564-7. 10.1056/NEJMc2004973 32182409 PMC7121658

[11] Siegel JD Rhinehart E Jackson M Chiarello L Health Care Infection Control Practices Advisory Committee 2007 guideline for isolation precautions: preventing transmission of infectious agents in health care settings. Am J Infect Control. 2007;35(10) Suppl 2;S65-164. 10.1016/j.ajic.2007.10.007 18068815 PMC7119119

[12] The Government of the Hong Kong Special Administrative Region. CHP investigates additional case of novel coronavirus infection. Hong Kong: The Government of the Hong Kong Special Administrative Region; 2020. Available from: https://www.info.gov.hk/gia/general/202002/01/P2020020100795.htm

[13] National Institute of Infectious Diseases (NIID). [Manual of laboratory diagnosis of novel coronavirus, Japan]. Tokyo: NIID. [Accessed 8 Mar 2020]. Japanese. Available: https://www.niid.go.jp/niid/ja/labo-manual.html#class0

[14] Moriarty LF Plucinski MM Marston BJ Kurbatova EV Knust B Murray EL Public Health Responses to COVID-19 Outbreaks on Cruise Ships - Worldwide, February-March 2020. MMWR Morb Mortal Wkly Rep. 2020;69(12):347-52. 10.15585/mmwr.mm6912e3 32214086 PMC7725517

[15] Nagasaki City. The outbreak situation of the prefecture in Nagasaki-shi of new coronavirus infectious disease. [Accessed 10 Jun 2020]. Available from: https://www.city.nagasaki.lg.jp.e.jc.hp.transer.com/fukushi/450000/454000/p034301.html

[16] Rothe C Schunk M Sothmann P Bretzel G Froeschl G Wallrauch C Transmission of 2019-nCoV infection from an asymptomatic contact in Germany. N Engl J Med. 2020;382(10):970-1. 10.1056/NEJMc2001468 32003551 PMC7120970

[17] Bai Y Yao L Wei T Tian F Jin D-Y Chen L Presumed asymptomatic carrier transmission of COVID-19. JAMA. 2020;323(14):1406. 10.1001/jama.2020.2565 32083643 PMC7042844

[18] World Health Organization (WHO). Q&A on coronaviruses (COVID-19). Geneva: WHO; 2020. Available from: https://www.who.int/news-room/q-a-detail/q-a-coronaviruses

[19] Guan W-J Ni Z-Y Hu Y Liang W-H Ou C-Q He J-X Clinical characteristics of coronavirus disease 2019 in China. N Engl J Med. 2020;382(18):1708-20. 10.1056/NEJMoa2002032 32109013 PMC7092819

[20] Hoehl S Rabenau H Berger A Kortenbusch M Cinatl J Bojkova D Evidence of SARS-CoV-2 infection in returning travelers from Wuhan, China. N Engl J Med. 2020;382(13):1278-80. 10.1056/NEJMc2001899 32069388 PMC7121749

[21] Zou L Ruan F Huang M Liang L Huang H Hong Z SARS-CoV-2 viral load in upper respiratory specimens of infected patients. N Engl J Med. 2020;382(12):1177-9. 10.1056/NEJMc2001737 32074444 PMC7121626

[22] Kigozi SP Kigozi RN Sserwanga A Nankabirwa JI Staedke SG Kamya MR Malaria burden through routine reporting: relationship between incidence and test positivity rates. Am J Trop Med Hyg. 2019;101(1):137-47. 10.4269/ajtmh.18-0901 31074412 PMC6609187

