# Authors’ correction for Euro Surveill. 2020;25(23)

**DOI:** 10.2807/1560-7917.ES.2020.25.24.2006181

**Published:** 2020-06-18

**Authors:** Eurosurveillance editorial team

**Affiliations:** 1 European Centre for Disease Prevention and Control (ECDC), Stockholm, Sweden

In the article ‘Descriptive study of COVID-19 outbreak among passengers and crew on Diamond Princess cruise ship, Yokohama Port, Japan, 20 January to 9 February 2020’ by Yamagishi et al. published on 11 June 2020, there was a mistake in the date the cruise ship left Yokohama Port, Japan. This was corrected on 12 June 2020, upon request of the authors.

